# A Green Resin Wood Adhesive from Synthetic Polyamide Crosslinking with Glyoxal

**DOI:** 10.3390/polym14142819

**Published:** 2022-07-11

**Authors:** Qianyu Zhang, Gaoxiang Xu, Antonio Pizzi, Hong Lei, Xuedong Xi, Guanben Du

**Affiliations:** 1 Yunnan Key Laboratory of Wood Adhesives and Glue Products, College of Material Science and Engineering, Southwest Forestry University, Kunming 650224, China; zqy20160922@163.com (Q.Z.); gaoxiangxu@swfu.edu.cn (G.X.); researcher0001@163.com (G.D.); 2 International Joint Research Center for Biomass Materials, Southwest Forestry University, Kunming 650224, China; 3 LERMAB, University of Lorraine, 88000 Epinal, France; antonio.pizzi@univ-lorraine.fr

**Keywords:** polyamide, wood adhesives, glyoxal, plywood, water resistance

## Abstract

Glyoxal is considered to be the most likely substitute for formaldehyde to synthesize resin adhesives for wood bonding due to its reactivity, structural characteristics, being non-toxic, low volatility, and acceptable cost. Regrettably, the performance of the resin synthesized using glyoxal to directly replace all formaldehyde is not totally satisfactory, especially as it has almost no water resistance. This makes such a simple alternative fail to be suitable for industrial production. To prepare an environment-friendly glyoxal-based adhesive with good bonding performance, the work presented here relies first on reacting citric acid and hexamethylene diamine, producing a polyamide, with glyoxal, and then crosslinking it, thus synthesizing a thermosetting resin (namely CHG) adhesive and applying it for plywood bonding. The plywood prepared exhibits excellent dry and wet shear strength, which are better than GB/T9846-2015 standard requirements (≥0.7 MPa), and even after being soaked in hot water at 63 °C for 3 h, its strength is still as high as 1.35 MPa. The CHG resin is then potentially an adhesive for industrial application for replacing UF (urea-formaldehyde) and MUF (melamine-urea-formaldehyde) adhesives for wood composites.

## 1. Introduction

Wood adhesives are critical components in the manufacture of wood-based products, such as particleboard, plywood panels, fiberboard, and other wood composites. They determine the performance of bonded products. Among the wood adhesives used, formaldehyde-based resins such as urea-formaldehyde (UF), phenol-formaldehylde (PF), melamine-formaldehyde (MF), and their co-condensed resin are the dominant resins [1,2,3]. However, their drawback is that formaldehyde has been classed as toxic and carcinogenic, and so is formaldehyde emission. Thus, for decades, research in the field of wood adhesives has focused on the improvement and modification of the synthesis of formaldehyde-based wood adhesives (for example UF resin) to improve their performance and reduce formaldehyde emission [4,5]. At the same time, the development of green adhesives has also drawn much attention and is strongly progressing, substantial research on this having already been carried out [3,6,7,8,9,10,11]. 

Using glyoxal to completely replace formaldehyde to synthesize glyoxal-based resin is an attractive approach to prepare more green adhesives. This is due to its features of non-volatility and low or no toxicity compared with formaldehyde [12]. Some research on the application of urea-glyoxal (UG) resin for plywood and particleboard has already been carried out, but because of the low condensation of UG, resulting in an undesirable performance, it is not adaptable to wood bonding [13]. The partial substitution of formaldehyde was then achieved by Deng et al. by a co-condensed urea-glyoxal-formaldehyde (UGF) resin with good bonding results for plywood, thus improving the degree of polymerization of the resin [14]. As this still entails the use of a much smaller proportion of formaldehyde, the problem of its emission persists. Consequently, melamine was selected to react with glyoxal to prepare a melamine-glyoxal (MG) resin, because its reactivity is higher than that of urea, and as it has a higher number of reactive sites, thus rendering it easier to obtain branched structures and an improved performance. Unfortunately, although such an MG resin has a branched structure and is used in particleboard preparation, the internal bond strength of the particleboard bonded with it having been effectively improved and complying with the standard requirements, the panels’ water absorption and thickness swelling were still not satisfactory [15]. By co-condensing melamine, glyoxal, and glutaraldehyde to obtain a melamine-glyoxal-glutaraldehyde (MGG’) resin and advantageously using ionic liquids as hardeners to decrease its energy of curing, the water resistance of the resin has been improved [16]. However, the high viscosity of this resin makes it suitable only for plywood production and not for particleboard and fiberboard, which is a great limitation.

In order to prepare a glyoxal-based resin with suitable viscosity with wide applicability to different wood composites, a polyamide prepolymer has been synthesized by reacting citric acid and hexamethylene diamine, then crosslinking this by condensing it with glyoxal to obtain a green resin adhesive, namely CHG. This is due to the amine and amide groups in the polyamide being able to react with the aldehyde groups by a Schiff reaction and polycondensation [17]. Polyamides are a universal plastic material and have been widely used in various industrial applications. The most common polyamide is nylon, extensively used for engineering plastics in machinery, automobiles, electrical appliances, textile equipment, chemical equipment, aviation, metallurgy, and other fields [18,19,20]. Polyamides are also used as adhesives or hot-melt adhesives, but these have relatively rarely been reported. In particular, polyamides are rarely reported as thermosetting adhesives for wood panels industrial production. The main reason for this is that general polyamide plastic products possess linear molecular structures and cannot form cured solids by thermal curing reactions [18,19]. Therefore, in the work presented here, citric acid was reacted with hexamethylenediamine to obtain a branched polyamide with thermosetting potential. Then, this polyamide was cross-linked with glyoxal to improve the branch abundance and to obtain a thermosetting resin usable as a wood adhesive. Various ratios of glyoxal to polyamide were tested in this work to prepare a CHG resin with outstanding bonding properties, and this resin can be suitable for the industrial manufacture of wood composites.

## 2. Materials and Methods

Citric acid (99.0%, Ar) and hexamethylene diamine (99.0%, Ar) are from Sinopharm Chemical Reagent Co., Ltd., Shanghai, China; Glyoxal 40 wt.% in water, Shanghai Zhanyun Chemical Co., Ltd., Shanghai, China.

### 2.1. CHG Adhesive Resins Preparation

**Figure 1:**
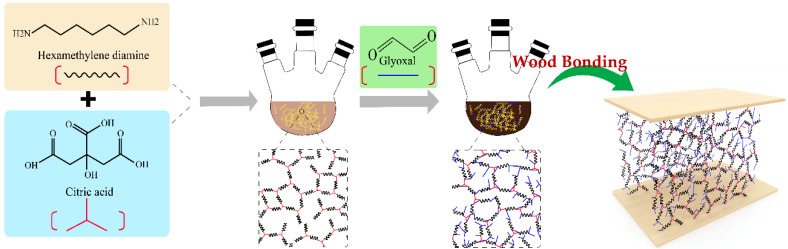
Schematic representation for the preparation of CHG resin and its using for wood bonding.

The CHG adhesive resins synthesis was as follows: 24.65 g of hexamethylene diamine (HDM) and 29.23 g of water were mixed in a flask at room temperature by magnetic stirring, and then 31.52 g of citric acid solid was added slowly; after this, the mixture was heated to 100 °C in an oil bath for 2 h, and then the temperature was reduced to 60 °C, and glyoxal was added for another 30 min reaction to obtain the CHG resin then to be used for plywood bonding(as shown in Figure 1). According to the previous work [21], the proportion of glyoxal was based on 0.6, 0.8, 1.0,1.2, 1.4, 1.8 and 2.2 times the molar mass of hexamethylene diamine, respectively. Correspondingly, the prepared resins were named CHG-0.6, CHG-0.8, CHG-1.0, CHG-1.2, CHG-1.4, CHG-1.8 and CHG-2.2. 

### 2.2. FTIR Spectrometry

Fourier transform infra-red (FTIR) analysis with a Shimadzu IRAffinity-1 spectrometer was used to confirm the relevant structures present. The reference spectrum used a potassium bromide tablet (ACS, ACROS Organics) as blank. An equivalent potassium bromide tablet was mixed with 5% *w*/*w* of the powdered samples for analysis. A 32-scan transmission spectrum at a 2.0 resolution was then obtained, and the wave number ranged between 400 and 4000 cm^−1^.

### 2.3. Characterization by ESI-MS (Electrospray Ionization Mass Spectrometry)

The preparation for analysis of the samples was performed by diluting with deionized water a 10 μL resin solution down to 1.0 mL. A quadrupole time-of-flight (Q-TOF) high-resolution mass spectrometer (Q-TOF liquid chromatography/mass spectrometry (LC/MS) 6540 series, Agilent Technologies, Santa Clara, CA, USA) coupled with electrospray ionization (ESI) was used for analysis. A Mass Hunter Workstation software was used for data acquisition. A positive ESI mode was used for detection. The optimized MS parameters were: voltage of the fragmentor set at 135 V, with the capillary at 3500 V and a 65 V setting for the skimmer; for drying (300 °C, 8 L min^−1^) and nebulizing (30 psi), nitrogen gas was employed.

### 2.4. Differential Scanning Calorimetry

Differential Scanning calorimetry was used in the work presented here for the CHG adhesives. The analysis was carried out with a DSC spectrometer (NETZSCH DSC 204 F1, Selb, Germany) at a 10 °C/min heating rate with the test temperature ranging between 30 °C and 250 °C, using a NETZSCH Proteus Analysis software(NETZSCH, Selb, Germany) for data analysis.

### 2.5. Dynamic Thermomechanical Analysis (DMA)

A NETZSCH DMA-242 thermal analyzer was used to test the CHG resins. Each adhesive sample was placed within a sandwich of 50 mm × 10 mm × 3 mm^3^ of two plies of poplar wood. The sandwiches so assembled were tested in the 30–250 °C range at a 10 °C/min heating rate. The test was carried out in non-isothermal three-point bending mode at a 10 Hz frequency with a 60µm strain amplitude and 1.5 N dynamic force. A NETZSCH Proteus software was used for data analysis.

### 2.6. Thermogravimetric Analysis (TGA)

Thermogravimetric analysis was used to test the thermal stability of the cured CHG adhesives in the 30 °C to 750 °C range at a 5 °C/min heating rate and under nitrogen atmosphere. The mass of each tested sample was 5.5 mg, and the TGA equipment used was a Q50 TGA (TA Instruments, New Castle, DE, USA).

### 2.7. Preparation and Testing of Plywood

Three-layer plywood panels bonded with CHG resins were prepared in the laboratory by a hot-pressing process using 180 °C temperature and 1.0 MPa pressure hot pressing for 5 min. The glue spread used was of 240 g/m^2^, and the thickness of poplar (*Populus tremuloides*) veneers used here was 2 mm. The dry shear strength and wet strength after 24 h soaking in cold water and 3 h in 63 °C hot water were tested to evaluate the performance of CHG adhesives. The prepared plywood samples were stored under conditions of 20 °C and 12% r.h. for 2 days; then, 10 samples were cut for measurement averaging and finally tested according to China National Standard GB/T 14074-2006 and GB/T17657-2013 [22,23]. The bond strength was determined from the shear strength according to the equation: shear strength (MPa) = maximum force (N)/bonded area (mm^2^).

## 3. Results and Discussion

### 3.1. LC-MS Analysis of CHG Resin

Soft ionization technique-based ESI-MS spectrometry shows directly the peaks of the molecular weights (MW) of the compounds formed, in particular, when these differ in MW [24]. The combination of FTIR analysis and chemical theory provision allows the deduction of the possible structures of the molecular species formed. The mass spectrometry results of the CHG-0.8 adhesive, shown in Supplementary Information Figure S1a–c, indicate the species obtained by reacting citric acid with hexamethylene diamine and then crosslinked with glyoxal. First, what cannot be avoided is the small molecular weight chemical species formed from the addition reaction of glyoxal with the remaining hexamethylenediamine, as can be observed for the peak at 174 Da. However, considerable evidence of the formation of the amide structures derived from the reaction of citric acid and hexamethylenediamine is obtained (as shown in Supplementary Information Table S1), certifying that the CH polyamide has been formed in the first stage of the preparation process. 

Many research works already testify that amide groups can react well with the aldehyde groups from glyoxal leading to Schiff bases [17,25,26, 27]. This is also confirmed by the CHG-0.8 results from LC-MS (in Supplementary Figure S1) with the assignment of the probable structures shown in Table S1. A predominant nucleophilic addition involving the CH polyamide resin with glyoxal appears to occur according to the structures determined for many of the oligomers formed, as indicated by the 349 Da and 504 Da peaks. Structural chain branching of the same type can also occur, as a number of the CH polyamide amide groups may well react with several glyoxal molecules, as, for example, for a 1061 Da structure, namely:

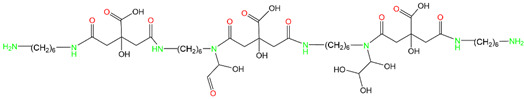



It is of considerable interest that such oligomers can continue reacting with the amide groups from another oligomer, being glyoxal a dialdehyde, as for the 1941 Da peak as following structure: 

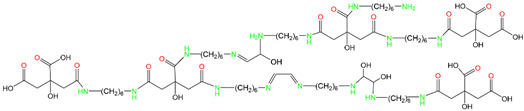



The cross-linking of the polyamide by glyoxal forms the final hardened tridimensional network of the resin. Coincidentally, structural branching of the oligomers formed ensures that the adhesive possesses excellent bonding properties [12].

Certainly, it needs to be clearly pointed out that a higher ratio of glyoxal addition of the CHG resin results in a more abundant branched structure, which is very easily predictable, although not all these CHG resins were analyzed by LC-MS. The rich branched structure of resin can ensure good bonding properties [12], which can be verified in the following bonding performance of resins. But it is not the case that the more glyoxal addition the better, as excess glyoxal may also lead to a decline in the bonding performance, which will be explained in the subsequent discussion. 

### 3.2. FTIR Analysis of CHGs

**Figure 2:**
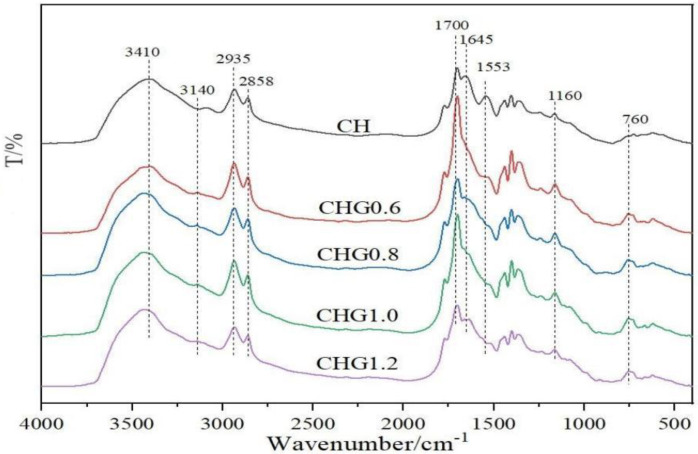
Infrared spectrum test results of the solid CH and CHG resins.

The FTIR spectrum of the solid CHG adhesives is shown in Figure 2. The O-H and N-H stretching vibrations are represented by the wide 3100–3600 cm^−1^ band. The C-H deformation is represented by both the 2930 and 2860 cm^−1^ bands. The peak at 1770 cm^−1^ is attributed to a C=O group from carboxyl that can be in the extremity of the CH molecule structures. C=O stretching is represented by the 1700 cm^−1^ band, and to the C=O stretching vibration of the -CO-NH- grouping is assigned the 1645 cm^−1^ peak, the shift of which to a lower wavenumber being due to the amino group influence [28]. The absorption peak of the N-H group appears at 1553 cm^−1^, which can be seen in the CH curve, while here, only a faint peak is observed in the CHG-0.6 resin. This is due to the Schiff base and polycondensation reaction between glyoxal and amino groups, so that it is consumed. As the proportion of glyoxal increases to 0.8 mol (curve CHG-0.8 in Figure 2) or more, the N-H stretching vibration peak has almost disappeared. It can be inferred from this that the synthesized CH polyamide and glyoxal react fully, thus consuming all or almost all the available –NH- groups, leading to more branched structures, thus ensuring a good bond strength [29]. The peak at 760 cm^−1^ belongs to the bending vibration of O-H bonds, and the evident strengthening of this peak can be observed by comparing the CHG resins to the CH polyamide. This occurs because of the formation of new hydroxyl groups during the reaction of glyoxal with the CH resin amide groups. It also contributes to the evidence that, in CHG resins, a high level of cross-linking has occurred due to the reaction of glyoxal with the CH polyamide.

### 3.3. Bonding Performance of Resins

**Figure 3:**
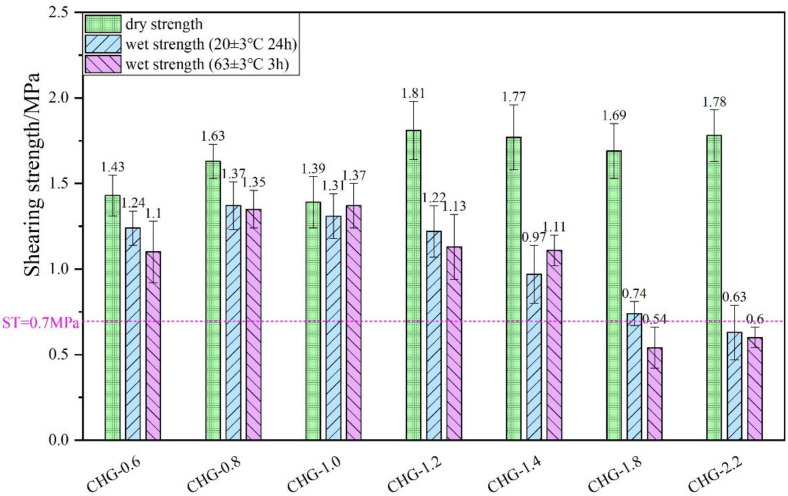
Bonding performance of plywood bonded with CHGs resins.

Figure 3 shows dry, 24 h cold water soaking and 3 h in 63 ± 2 °C shear strengths of the CHG-bonded plywood panels. First, Figure 3 shows the totality of the CHG adhesives, showing a good dry shearing strength, as they are all higher than the standard requirement of China National Standard GB/T9846-2015 (≥0.7 MPa), which is suitable for CHG resins in all proportions in this work. More satisfying is the wet strength of the plywood test results. Both 24 h cold water soaking strength and 3 h 63 °C hot water strength of the boards bonded by CHG resins with a proportion of glyoxal from 0.6 to 1.4 satisfy the standard requirement of 0.7 MPa or higher. With the progressive increase in the proportion of glyoxal, the bonding performance of the synthesized resin, especially the plywood wet shear strength, shows a weakening trend, which may be caused by excessive addition of glyoxal. This is because the linear oligomerization with hydrophilic group hydroxyls can be formed from the glyoxal by aldol condensation, which reduces the water resistance of the resin [12]. Regardless, the results of all CHG resins are clearly better than the widely used urea-formaldehyde (UF) adhesives with regard to bonding strength, especially for water resistance, judging from the 24 h cold water or 63 °C hot water wet shear strength test results. 

### 3.4. DMA Analysis of CHGs

**Scheme 1:**
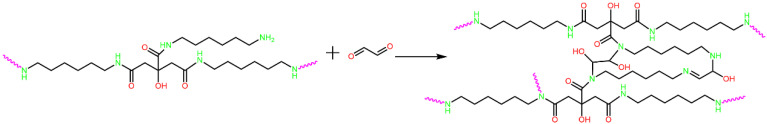
Example of glyoxal crosslinking reaction with polyamide.

**Figure 4:**
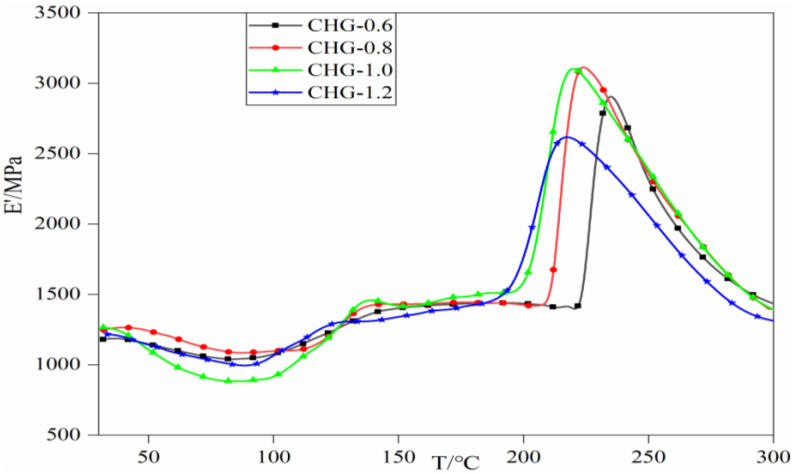
DMA results of the CHG resin adhesives.

The plywood bonding performance test results attest that too much glyoxal reduces the properties of the synthesized CHG resins. Thus, dynamic thermomechanical analysis was only used to test four CHG resins prepared by glyoxal additions of 0.6, 0.8, 1.0, and 1.2, the results being shown in Figure 4. Intuitively, all curing by DMA of CHG resins show an increase in Young’s modulus as a function of an increase in temperature, starting at around 200 °C. This means that CHG resin can begin to cure quickly at this temperature and provide bond strength to the test specimen, but it also indicates that CHG resins require a higher curing temperature. Comparing the four curves in Figure 4, it is indicated that, from CHG-0.6 to CHG-1.2, as the proportion of glyoxal increases, the initial curing temperature of the CHG resin decreases. Because of the high reactivity between glyoxal and the CH polyamide (as the reaction shown in Scheme 1), increasing the ratio of glyoxal makes the cross-linking reaction more effective and is thus sufficient to reduce the initial curing temperature of CHG resins. 

The strength of the test specimen can be reflected by the Young’s modulus in the DMA result, this correlation having been shown already several times in the literature [29,30,31]; thus, the Young’s modulus, in particular, its peak value, is an important object of investigation by DMA analysis. Apparently, CHG-0.8 and CHG-1.0 reflect higher modulus values than the other two resins, this being consistent with the result that CHG-0.8 and CHG-1.0 have a better bond strength than CHG-0.6 and CHG-1.2 in the plywood test results. In addition, it is also evident from the DMA curves that the curing temperature to be used for such CHG adhesives is relatively higher than that commonly used today for equivalent panels bonded with traditional adhesives such as urea-formaldehyde resin and phenol-formaldehyde resin [32,33]. 

### 3.5. DSC Analysis CHGs

**Scheme 2:**
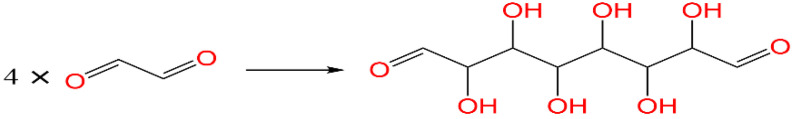
Example of glyoxal aldol condensation.

**Figure 5:**
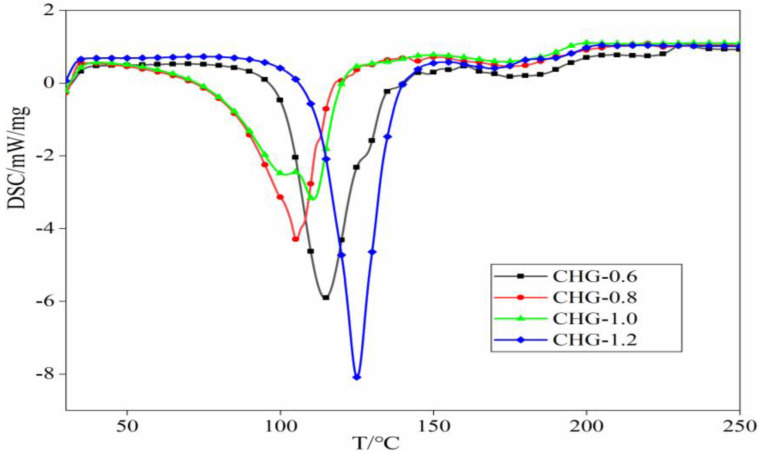
DSC results of the CHG resin adhesives.

In the present research work, DSC has also been used to analyze the thermal curing behavior of CHG resins, with the test results shown in Figure 5. Comparing the curves of CHG-0.6 and CHG-0.8, it can be observed that the peak resins’ curing temperatures are 118 °C and 105 °C, respectively, which means that the curing temperature of a CHG resin will decrease with the increase in the proportion of the glyoxal used. However, as the ratio of glyoxal increases to 1.0 to obtain the CHG-1.0 resin, the glyoxal level will be excessive in the resin, making the curing temperature of CHG-1.0 higher than that of CHG-0.8, although the increase is not so significant. Further increases in the proportion of glyoxal to obtain CHG-1.2 resin raise its curing temperature to 127 °C, which may be due to the aldol condensation of the glyoxal in excess (Scheme 2) [12]. These formed aldol-derived oligomers possess lower reactivity with CH molecules, thus leading to an increase in the curing temperature of the entire resin system. Moreover, aldol condensation also produced linear oligomeric hydroxyls with a hydrophilic group; these substances reduced the water resistance of the resin. 

In general, as the proportion of glyoxal increases, the curing temperature trend of the synthetic CHG resins changes from an increase to a decrease. This is one of the explanations for the change in the performance of the adhesive.

### 3.6. TG Analysis CHG Resins

**Figure 6:**
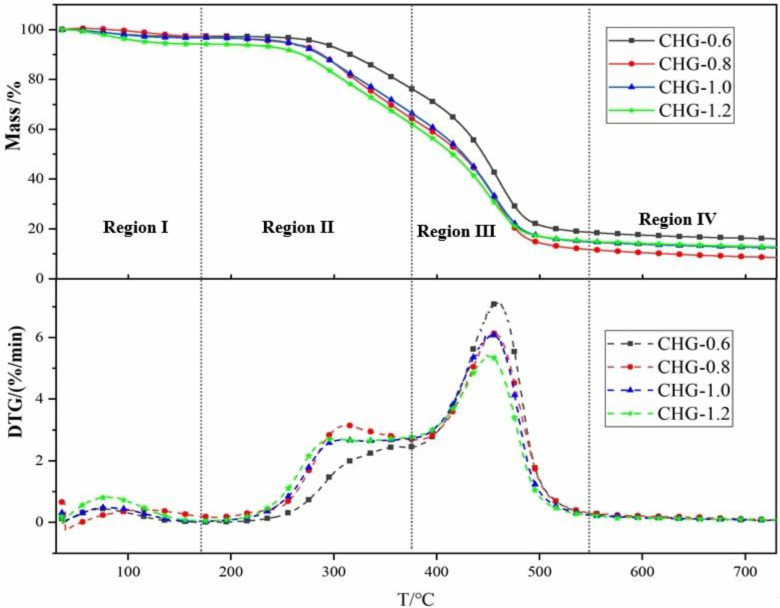
TGA results of the CHG resin adhesives.

In order to evaluate the thermal stability of the synthesized green CHG-0.6, CHG-0.8, CHG-1.0, and CHG-1.2 cured resins, they were tested by thermogravimetric analysis, and the results are shown in Figure 6. The weight loss as a function of the increase in temperature of the four resins evaluated is similar. The inference of this observation is that, notwithstanding the molar ratio differences in glyoxal in the resin formulations, similar reaction products are obtained. A rather limited weight loss is observed in the 30–150 °C range representing Region I. This loss should be due to the elimination/evaporation of water and of lower molecular weight compounds, and the water lost being adsorbed on the surface of the solid resin powder [34]. The 150–380 °C range named as Region II is attributed to the dehydration of the hydroxyl groups formed during the reaction of glyoxal with the CH amide groups. Region III is the stage in which the main loss occurs and thus where the adhesive cross-linked network is destroyed due to the cleavage of the polymer chains and their decomposition [24,34,35]. The final remaining solid in the region IV is charcoal, with values in CHG-0.6, CHG-0.8, CHG-1.0 and CHG-1.2 of 16.5%, 9%, 12.6% and 13%, respectively. The solid CHG-0.8 resin has the least residual carbon, and thus the glyoxal has the most complete reaction with the CH polyamide. This results in a higher proportion of hydrogen, oxygen and nitrogen in the resin solids, thus causing the gasification of N, H and O or water lost during network cracking.

## 4. Conclusions

This work presents a new glyoxal-based green adhesive used for wood bonding by co-reacting citric acid, hexamethylenediamine and glyoxal to synthesize CHG resins. Since no formaldehyde was used throughout the preparation process, CHG can be considered an environmentally friendly adhesive. LC-MS and FTIR results confirm the cross-linking reaction between glyoxal and the synthetic polyamide, and good thermal stability of this obtained resin can also be observed by TGA analysis. The CHG resin exhibited a good water and moisture resistance and equally remarkable bond strength, when glyoxal’s molar ratio to hexamethylene diamine is 0.8:1. CHG bonded plywood dry strength, with 24 h cold water and 63 °C hot water soak wet strengths of 1.63 MPa, 1.37 MPa and 1.35 MPa, respectively, thus satisfying the requirement of the China National Standard GB/T 9846-2015 (≥0.7 MPa), and indicates that such a resin is greatly competitive as a substitute for the existing UF resin adhesives.

## Data Availability

Not applicable.

## Supplementary Materials

The following supporting information can be downloaded at: https://www.mdpi.com/article/10.3390/polym14142819/s1. Table S1. Oligomers identified by LC-MS mass spectrometry of the CHG-0.8 resin; Figure S1. LC-MS results of CHG-0.8 resin (a–c).
