# Supplementary material for: A Green Resin Wood Adhesive from Synthetic Polyamide Crosslinking with Glyoxal
Source: Polymers (Basel). 2022 Jul 11;14(14):2819. doi: 10.3390/polym14142819 (PMC9318107; doi:10.3390/polym14142819)
Supplement: Supplementary file 1 [file polymers-14-02819-s001.zip › polymers-1750213-supplementary.pdf]

Supplementary Information

A Green Resin Wood Adhesive from Synthetic Polyamide Crosslinking with Glyoxal

Qianyu Zhang[1,2], Gaoxiang Xu, Antonio Pizzi[3], Hong LEI*[1,2], Xuedong XI*[1,2], Guanben DU[1,2]

1, Yunnan key laboratory of wood adhesives and glue products, College of Material science and Engineering, Southwest Forestry University, 650224 Kunming, China;

2, International Joint Research Center for Biomass materials, Southwest Forestry University, 650224 Kunming, China;

3, LERMAB, University of Lorraine, 88000 Epinal, France;

Corresponding Author

E-mail addresses: leihong@swfu.edu.cn (H. Lei), xuedong.xi@swfu.edu.cn (X. Xi).

Table S1. Oligomers identified by LC-MS mass spectrometry of the CHG-0.8 resin.

M/Z	Structure
174Da	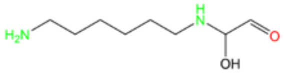
349Da	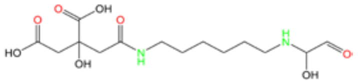
443Da	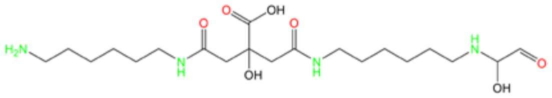
504Da	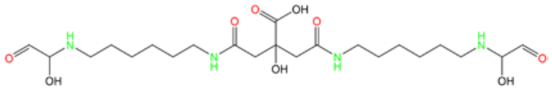
938Da	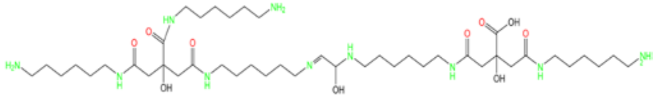 with Na^+
978Da	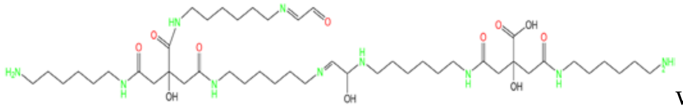 with Na^+
1061Da	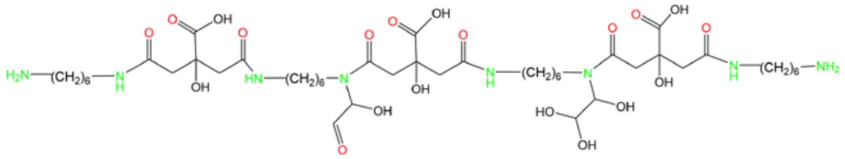

1543Da	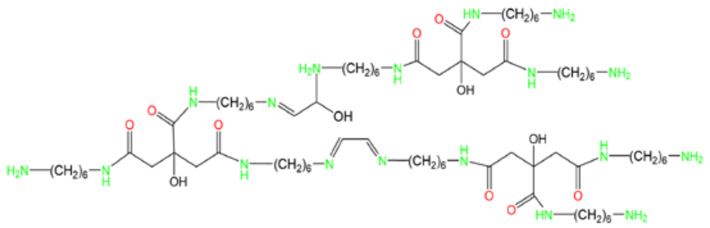 with Na+
1941Da	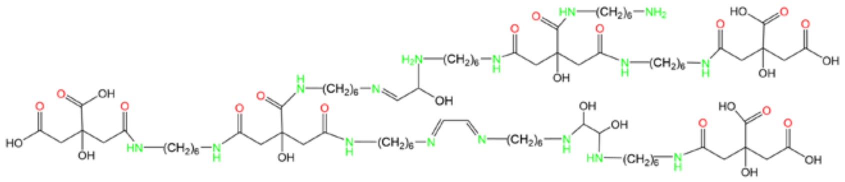

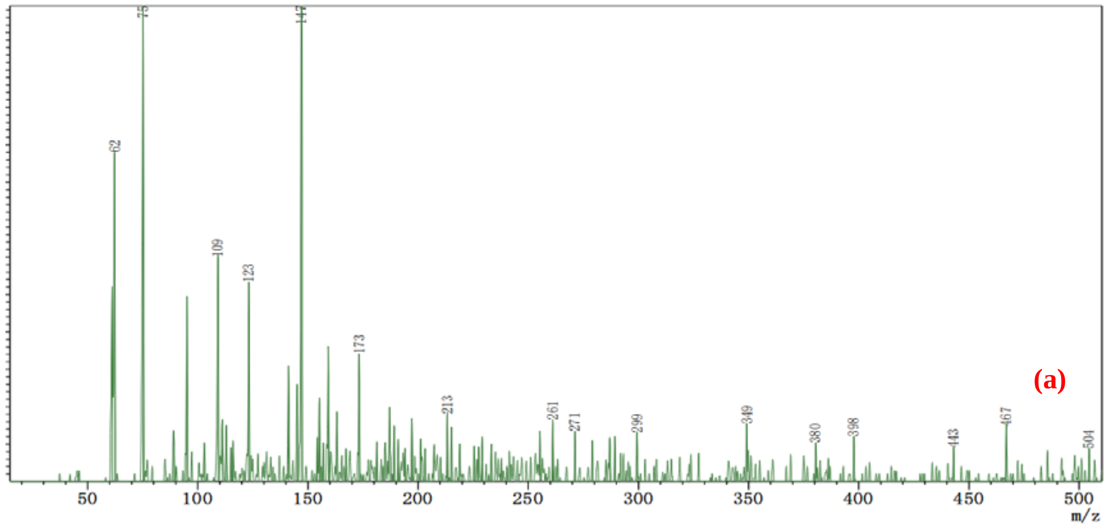

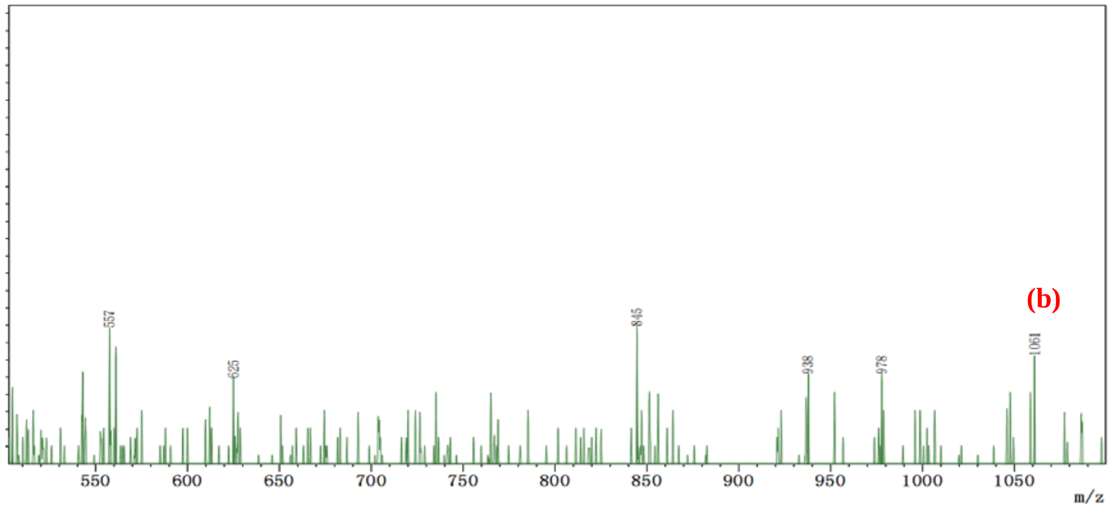

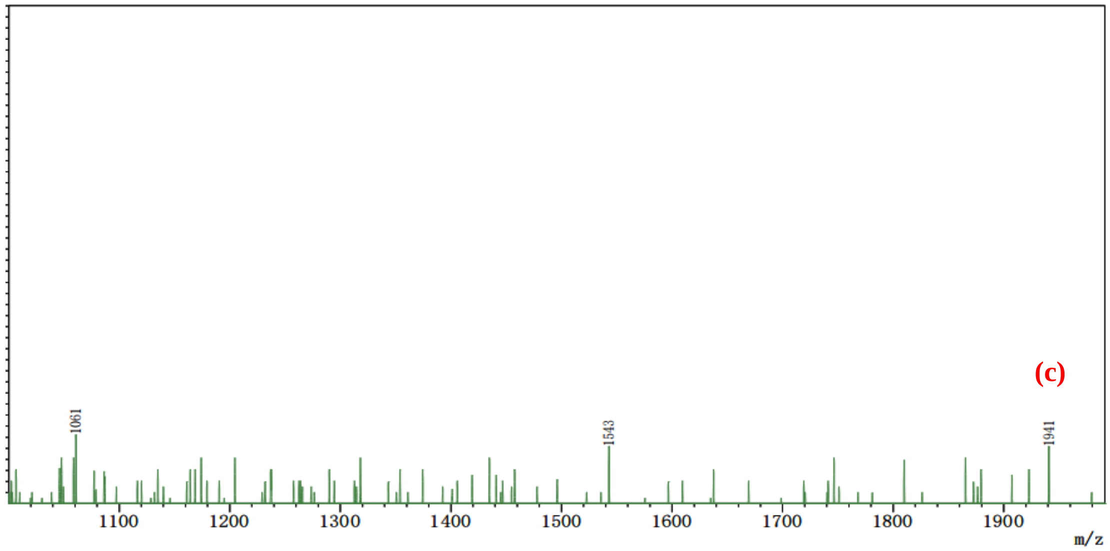

Figure S1. LC-MS results of CHG-0.8 resin(a-c)
